# The effect of changes in intraocular pressure on the risk of primary open-angle glaucoma in patients with ocular hypertension: an application of latent class analysis

**DOI:** 10.1186/1471-2288-12-151

**Published:** 2012-10-04

**Authors:** Feng Gao, J Philip Miller, Stefano Miglior, Julia A Beiser, Valter Torri, Michael A Kass, Mae O Gordon

**Affiliations:** 1 Division of Biostatistics, Washington University School of Medicine, St. Louis, MO, 63110, USA; 2 Department of Ophthalmology & Visual Sciences, Washington University School of Medicine, St. Louis, MO, 63110, USA; 3 Department of Ophthalmology, The Policlinico di Monza, University Bicocca of Milan, Milan, Italy; 4 Laboratory of New Drugs Development Strategies, Mario Negri Institute, Milan, Italy

**Keywords:** Latent class analysis, Longitudinal data, Time-dependent covariate, Prediction model, Survival data, Primary open-angle glaucoma, Intraocular pressure fluctuation

## Abstract

**Background:**

Primary open-angle glaucoma (POAG) is one of the leading causes of blindness in the United States and worldwide. While lowering intraocular pressure (IOP) has been proven to be effective in delaying or preventing the onset of POAG in many large-scale prospective studies, one of the recent hot topics in glaucoma research is the effect of IOP fluctuation (IOP lability) on the risk of developing POAG in treated and untreated subjects.

**Method:**

In this paper, we analyzed data from the Ocular Hypertension Treatment Study (OHTS) and the European Glaucoma Prevention Study (EGPS) for subjects who had at least 2 IOP measurements after randomization prior to POAG diagnosis. We assessed the interrelationships among the baseline covariates, the changes of post-randomization IOP over time, and the risk of developing POAG, using a latent class analysis (LCA) which allows us to identify distinct patterns (latent classes) of IOP trajectories.

**Result:**

The IOP change in OHTS was best described by 6 latent classes differentiated primarily by the mean IOP levels during follow-up. Subjects with high post-randomization mean IOP level and/or large variability were more likely to develop POAG. Five baseline factors were found to be significantly predictive of the IOP classification in OHTS: treatment assignment, baseline IOP, gender, race, and history of hypertension. In separate analyses of EGPS, LCA identified different patterns of IOP change from those in OHTS, but confirmed that subjects with high mean level and large variability were at high risk to develop POAG.

**Conclusion:**

LCA provides a useful tool to assess the impact of post-randomization IOP level and fluctuation on the risk of developing POAG in patients with ocular hypertension. The incorporation of post-randomization IOP can improve the overall predictive ability of the original model that included only baseline risk factors.

## Background

Ocular hypertension is a leading risk factor for the development of primary open-angle glaucoma (POAG) which remains one of the major causes of blindness in the United States and worldwide [1-5]. It is estimated that approximately 4% - 7% of the population over the age of 40 years have ocular hypertension without detectable glaucomatous damage using standard clinical tests, and thus as many as 3 to 6 million Americans are at risk for developing glaucoma because of ocular hypertension [6-8]. Intraocular pressure (IOP) is the only known modifiable risk factor for POAG. Lowering the level of IOP has been shown to effectively delay or prevent glaucomatous visual damage in different phases of disease progression by many large-scale multicenter clinical trials, including the Ocular Hypertension Treatment Study (OHTS) [9], the Early Manifest Glaucoma Trial [10], and the Advanced Glaucoma Intervention Study [11].

In recent years, one of the hot topics in glaucoma research has been the effect of IOP fluctuation (IOP lability), both within a single day (short-term fluctuation) and from visit to visit (long-term fluctuation) on POAG [12,13]. Measures of IOP fluctuation have included a wide range of quantities - peak, trough, variance, and range, etc. [13] However, since subjects with high mean IOP often show large IOP variability over time, it is challenging to disentangle the effect of fluctuation from mean IOP. A recently emerged technique for longitudinal data analysis, latent class analysis (LCA) [14], provides an appealing approach to this question. Rather than dealing with individual measures of fluctuation, LCA identifies distinct patterns of longitudinal profiles based on the combination of summary statistics (i.e., mean level and variability) and hence provides information complementary to the conventional methods. LCA uses the patterns of serial biomarker readings available for subjects, together with baseline covariates and disease outcomes, to divide subjects into a number of mutually exclusive subpopulations (classes). The class membership is unobserved (latent) and determined by the class-specific parameters in a data-driven basis.

In this paper, we used LCA to model the post-randomization IOP in the OHTS. For each class, the change of IOP was characterized by 4 parameters: the initial IOP level (I), the linear (L) and quadratic (Q) trend over time, and the variance of IOP (V). We used data from the European Glaucoma Prevention Study (EGPS) [15], another large-scale multicenter randomized clinical trial of patients with ocular hypertension, for external independent validation. We first fit an unconditional (without any covariates) LCA to determine the optimal number of distinct patterns that best described the IOP change for each study. Then a conditional model was constructed by adding baseline covariates as the antecedents (predictors) of IOP change and time to POAG as a consequence (outcome) of IOP change [16]. This analysis enhanced our understanding of the interrelationships among the IOP change, the baseline covariates, and the risk of developing POAG. This also provided evidence towards our ultimate goal to improve the prediction of POAG in patients with ocular hypertension.

## Methods

### Study cohort

Our study used data from OHTS and EGPS, the two largest randomized trials to test safety and efficacy of topical hypotensive medication in preventing the development of POAG. In OHTS, 1636 subjects were randomized to either observation or treatment with ocular hypotensive medication and followed for a median of 78 months [9]. In EGPS, 1077 subjects were randomized to either placebo or an active treatment (dorzolamide) and followed for a median of 55 months [15]. The two studies shared many key similarities in the study protocol and generated data of high quality. In both studies, for example, the outcome ascertainment was performed by specialized resource centers where readers were masked as to randomization assignment and information about the participant’s clinical status, and the attribution of abnormality due to POAG was performed by a masked Endpoint Committee. Detailed information on the similarity and discrepancy between OHTS and EGPS as described by Gordon et al. [17]. This study was approved by the Institutional Review Boards of Washington University in St. Louis and the University Bicocca of Milan.

In this paper, we excluded IOP values measured after POAG onset. The primary endpoint was time from randomization to the development of POAG. Those subjects who did not develop POAG were censored at the date of study closeout. In addition to the follow-up data, following 13 demographic and clinical characteristics at randomization were also included in this paper: treatment assignment (TRT, 0 for observation/placebo and 1 for treatment), male gender (Male), black race (Black), age at randomization (Age, decade), baseline IOP (IOP0, mmHg), central corneal thickness (CCT, μm), pattern standard deviation (PSD, dB), vertical cup/disc ratio (VCD), the use of systematic beta blocker (BB) or Calcium channel blockers (CHB), and the history of diabetes (DM), heart diseases (Heart), or hypertension (HBP). These baseline factors were identified *a priori* as possible predictors for the development of POAG during the planning phase of the OHTS [18]. We excluded 34 subjects from EGPS with pigment dispersion and exfoliation syndromes (an exclusion criterion in OHTS). We also excluded subjects without any follow-up data (18 in OHTS and 47 in EGPS) or those with only 1 follow-up visit (19 in OHTS and 25 in EGPS). Therefore, these subjects with at least 2 follow-up visits (1600 from OHTS and 971 from EGPS) constituted our study cohort for the unconditional LCA. In the conditional LCA, we further excluded subjects without CCT measurements (169 in OHTS and 143 in EGPS) or those with missing values in any other baseline factors (6 in EGPS). Table 1 presented the summary statistics of baseline covariates and post-randomization data for each study, where the binary data were summarized as counts and proportions, while the continuous variables were summarized in means and standard deviations (SD). For consistency with previous analyses [17,18], values for the baseline eye-specific variables (CCT, PSD, VCD, and baseline IOP) for each participant were the average of two eyes (with the exception of the EGPS participants with only one eye eligible for the study). For the post-randomization IOP, however, only eye-specific data were used because averaging two eyes could underestimate the true intra-patient IOP variability. We took advantage of the fact that IOPs between two eyes were highly correlated (with an intra-class correlation coefficient of 0.75), and follow-up IOPs were chosen from the first eye developed POAG or an eye selected randomly in participants without POAG. Since the continuous baseline covariates were measured in quite different scales, they were standardized to have mean 0 and variance 1 throughout the remainder of this paper. As such, for these variables the odds ratios (OR) and hazard ratios (HR) from the regression models represented the effect per 1-SD change.

**Table 1:** Summary statistics of baseline predictors and follow-up data for OHTS and EGPS, where categorical variables are summarized as counts and proportions, while the continuous variables are summarized in means and standard deviations (SD).

**Variables**	**OHTS (N = 1600)**	**EGPS (N = 971)**
**Baseline predictors**		
TRT	795 (49.7%)	487 (50.2%)
Male	687 (42.9%)	445 (45.8%)
Black	396 (24.8%)	1 (0.1%)
AGE (decades)	5.56 (0.96)	5.70 (1.02)
IOP0 (mmHg)	24.9 (2.69)	23.4 (1.62)
CCT (μm)	572.6 (38.5)	573.3 (37.5)
PSD (dB)	1.91 (0.21)	2.00 (0.52)
VCD	0.39 (0.19)	0.32 (0.14)
BB	71 (4.4%)	64 (6.6%)
CHB	190 (11.9%)	66 (6.8%)
DM	188 (11.8%)	55 (5.7%)
Heart	99 (6.2%)	109 (11.2%)
HBP	606 (37.9%)	279 (28.7%)
**Post-randomization IOP**		
Mean (mmHg)	21.44 (3.45)	19.73 (2.57)
SD (mmHg)	2.27 (1.04)	2.22 (1.03)
Median **#**visits (min-max)	13 (2–16)	9 (2–10)
**POAG**	146 (9.1%)	107 (11.0%)

### Statistical analysis

#### Unconditional LCA

Suppose there were *N* subjects and each subject had *n*_*i*_ pre-POAG IOP measures. Let *Y*_*i*_ *= {Y1, Y2, ……}* denote the post-randomization IOP and C_*i*_ represent the latent class membership of *i*^th^ individual, and θ_g_ be the vector of class-specific parameters that differentiate the G latent classes, with *i =1, 2, …,N,* and *g =1, 2, …,G,* respectively*.* Then the distribution of *Y*_*i*_ was a mixture distribution defined as [14],

(1) $$f\left(Y_{i}\right)=\sum_{g=1}^{G}\left\{\mathrm{Pr}\left(C_{i}=g\right)\cdot f\left(Y_{i}|C_{i}=g;\theta_{g}\right)\right\}$$

where $\mathrm{Pr}\left(C_{i}=g\right)$ represented the size (mixing proportion) of *g*^th^ latent class in the mixture and $f\left(Y_{i}|C_{i}=g;\theta_{g}\right)$ was the class-specific distribution of *Y*_*i*_ as detailed below.

**Figure 1:**
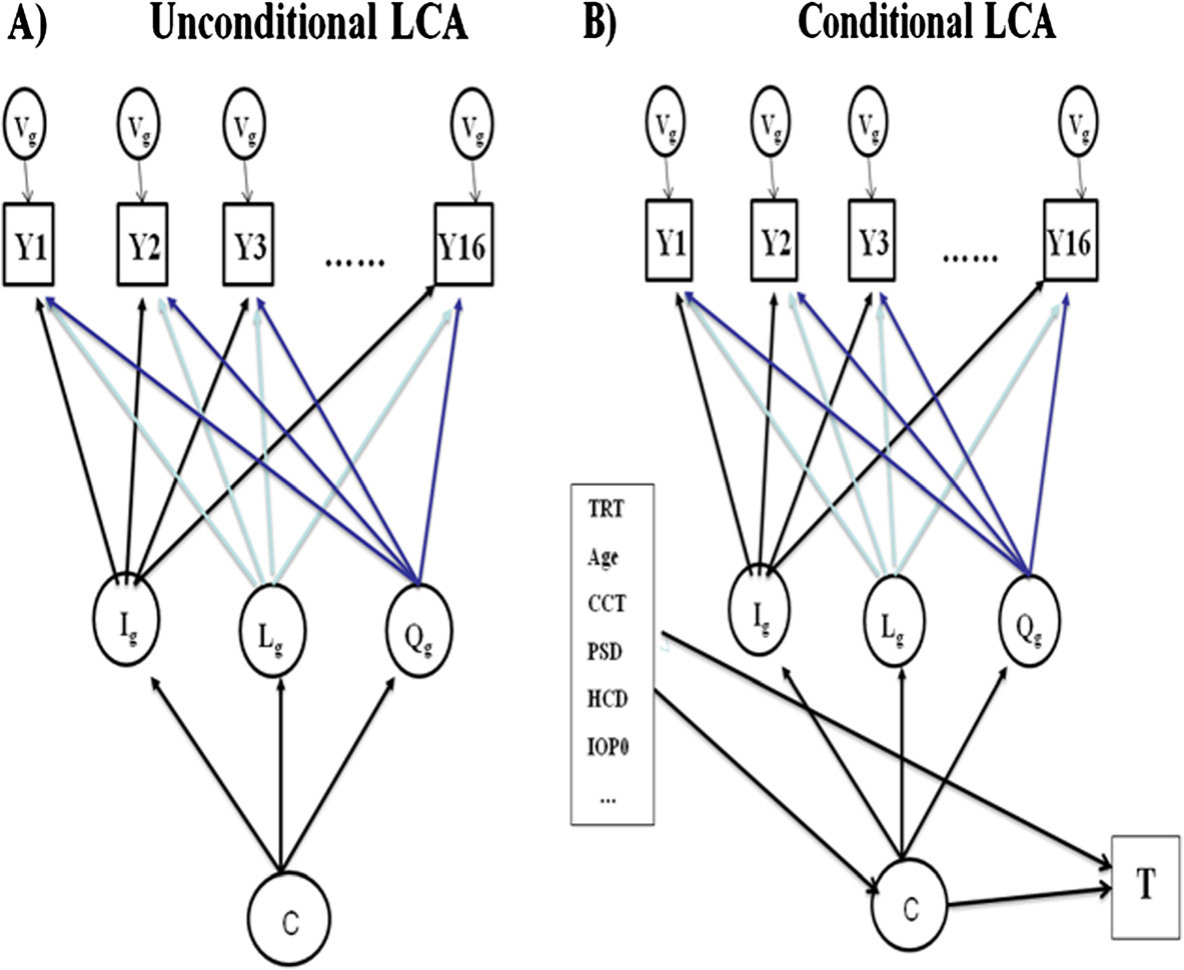
**Diagrams for unconditional (1A) and conditional (1B) latent class analysis (LCA) for OHTS data, where C denoted the latent classes.** The trajectory of post-randomization IOP (Y) in each class was described by 4 class-specific parameters: the initial IOP level (I), the systematic linear (L) and quadratic (Q) trend over time, and the variance of IOP (V).

• The mixing probability $\mathrm{Pr}\left(C_{i}=g\right)$ was modeled as a multinomial logistic regression, $\mathrm{Pr}\left(C_{i}=g\right)=\frac{\exp\left(\alpha_{0g}\right)}{\sum_{h=1}^{G}\exp\left(\alpha_{0g}\right)}$, where α_0g_ represented the log odds of membership in the *g*^th^ class relative to a reference class (class 1, say), with the parameter in the reference being 0 for identification.

• The specification of $f\left(Y_{i}|C_{i}=g;\theta_{g}\right)$ was aided by our previous experience on the joint modeling of longitudinal IOP and time to POAG in OHTS [19]. The joint model identified IOP variability as an independent predictor for POAG and also revealed that the IOP change can be better fit by a quadratic functional form. Therefore, we set $f\left(Y_{i}|C_{i}=g;\theta_{g}\right)=I_{g}+L_{g}t_{i}+Q_{g}t_{i}^{2}+\varepsilon_{i}$_,_ with $\varepsilon_{i}{\text{\textasciitilde{}N(0, V}}_{\text{g}})\;\text{and}\;\theta_{g}=\left\{I_{g},L_{g},Q_{g},V_{g}\right\}.$ Because high IOP was an eligibility criterion in both OHTS and EGPS, the estimated initial level (intercept *I*_g_) may be influenced by “regression to the mean”. To address this concern, we re-set the time 0 and the intercept was actually estimated at 1-year after randomization. We also assumed that follow-up IOPs were measured regularly every 6 months according to the protocol, i.e., with timing t_i_ = {−0.5, 0, 0.5, 1, …}. Figure 1A showed the diagram of an unconditional LCA for the OHTS data.

• Given the estimated parameters ${\overset{⌢}{\theta }}_{g}$ and the observed IOP, each individual can be assigned to the most likely class based on the probability of class membership (often termed as *posterior class probability*) [14],

• 

(2) $$p_{\mathrm{ig}}=\frac{\overset{⌢}{P}r\left(C_{i}=g\right)\cdot f(Y_{i}|C_{i}=g;{\overset{⌢}{\theta }}_{g})}{\sum_{h=1}^{G}\{\overset{⌢}{P}r\left(C_{i}=h\right)\cdot f\left(Y_{i}|C_{i}=h;{\overset{⌢}{\theta }}_{h}\right)\}}\text{.}$$

The best unconditional LCA was selected by enumerating and comparing a set of competing models differing only in the number of classes. In this paper, the model comparison was based primarily on the log likelihood values, including the Bayesian Information Criteria (BIC, with a smaller BIC indicating a better fit) and the Lo-Mendell-Rubin adjusted likelihood ratio test (LMR-LRT) [20]. A significant test of LMR-LRT indicated that the model with G-1 classes should be rejected in favor of the G-class LCA. In addition to the above statistical criteria, we also specified a minimum size for each class (with at least 5% participants in OHTS or 10% participants in EGPS) to ensure reliable within-class estimation. Once an optimal LCA was developed, a bootstrap method was used to assess whether patients with different patterns of IOP change have different susceptibility to POAG. Specifically, a class membership was generated for each individual from a multinomial distribution using the *posterior class probability*, and then a Cox model was fit to assess the effects of latent classes on POAG. Summary statistics such as hazard ratios and their 95% confidence intervals were estimated by repeating the above procedure 1,000 times.

#### Conditional LCA

Since patterns of IOP change were found to be associated with the risk of POAG in an unconditional LCA, a conditional LCA was built by adding baseline covariates as predictors to the IOP change and adding time to POAG as an outcome due to IOP change (Figure 1B). Let X_*i*_ denote the baseline predictors for *i*^th^ subject and *T*_*i*_ *= minimum(D*_*i*_*, U*_*i*_*)* be the observed time, where *D*_*i*_ was the time to POAG and U_*i*_ represented the censoring time independent of *D*_*i*_ . Let *Δ*_*i*_ be the corresponding event indicator, with *Δ*_*i*_ = 1 if POAG is observed and *Δ*_*i*_ = 0 otherwise. Let α and β denote effects of baseline covariates X_*i*_ on the IOP change and time to POAG respectively. Then the joint distribution of (*Y*_*i*_*, T*_*i*_) was a mixture distribution defined as [21],

(3) $$\begin{array}{l} f\left(Y_{i},T_{i}\right)=\sum_{g=1}^{G}\{\mathrm{Pr}\left(C_{i}=g;\alpha_{g}\right)\cdot f(Y_{i}|C_{i}=g;\theta_{g})\\ \;•\lambda {\left(T_{i}|C_{i}=g;\beta \right)}^{\mathrm{\Delta i}}\cdot S(T_{i}|C_{i}=g;\beta )\} \end{array}$$

• Similar to Model (1), the term $\mathrm{Pr}\left(C_{i}=g;\alpha_{g}\right)=\frac{\exp\left(\alpha_{0g}+\alpha_{g}X_{i}\right)}{\sum_{h=1}^{G}\exp(\alpha_{0h}+\alpha_{h}X_{i})}$ represented the size of *g*^th^ class in the mixture distribution and $f\left(Y_{i}|C_{i}=g;\theta_{g}\right)$ described the within-class IOP change.

• The term $\lambda \left(T_{i}|C_{i}=g;\beta \right)=\lambda_{0g}(\text{t)}\cdot \text{exp}\left(\beta X_{i}\right)$ described the risk of developing POAG in *g*^th^ class and $S(T_{i}|C_{i}=g;\beta )=\exp(-\int \lambda_{0g}(\text{t)}\cdot \text{exp}(\beta X_{i}(\text{dt})$ was the corresponding cumulative POAG-free probability, where λ_0g_(t) was the class-specific baseline hazard with all covariates being 0. In this paper, λ_0g_(t) was approximated by a piece-wise step-function with a 6-month interval. Following the conventional practice in joint latent class modeling [21,22], we assumed that the association between IOP change and time to POAG was introduced exclusively via λ_0g_(t), so that the longitudinal process and survival process were completely independent given the class membership. Therefore, neither time-dependent IOP values nor random effects of IOP were included in the survival function. We also assumed that the effects of covariates on POAG were common across latent classes.

The conditional LCA facilitated a better understanding of ocular hypotensive treatment on the risk of developing POAG. This model allowed us to determine whether the predictive accuracy on POAG can be improved by adding post-randomization IOP. For example, the survival probability (cumulative POAG-free rate) at any time t can be readily calculated as the average of the class-specific survival weighted by the posterior class probabilities,

(4) $$S(T_{i}=t)=\sum_{g=1}^{G}{\hat{p}}_{\mathrm{ig}}\cdot \hat{S}(T_{i}=t|C_{i}=g;\hat{\beta })$$

(5) $$\text{with}\;{\hat{p}}_{\mathrm{ig}}=\frac{\overset{⌢}{P}r\left(C_{i}=g;{\hat{\alpha }}_{g}\right)\cdot f(Y_{i}|C_{i}=g;{\overset{⌢}{\theta }}_{g})}{\sum_{h=1}^{G}\{\overset{⌢}{P}r\left(C_{i}=h;{\hat{\alpha }}_{h}\right)\cdot f\left(Y_{i}|C_{i}=h;{\overset{⌢}{\theta }}_{h}\right)\}},\;$$

(6) $$\begin{array}{l} \;\text{and}\;\hat{S}(T_{i}=t|C_{i}=g;\hat{\beta })\\ \;=\exp(-\int_{s=0}^{t}{\hat{\lambda }}_{0g}(\text{s})\cdot \text{exp}\left(\hat{\beta }X_{i}\right)\text{ds})\text{,} \end{array}$$

where $({\hat{\theta }}_{g},{\hat{\alpha }}_{g},\hat{\beta },\text{and}\;{\hat{\lambda }}_{0g}(\text{t}))$ were the estimated parameters from the conditional LCA. In this paper, the parameter estimation for LCA was implemented using statistical package Mplus [23], while all the other analyses were performed using statistical package R [24].

## Results

### Unconditional LCA

**Figure 2:**
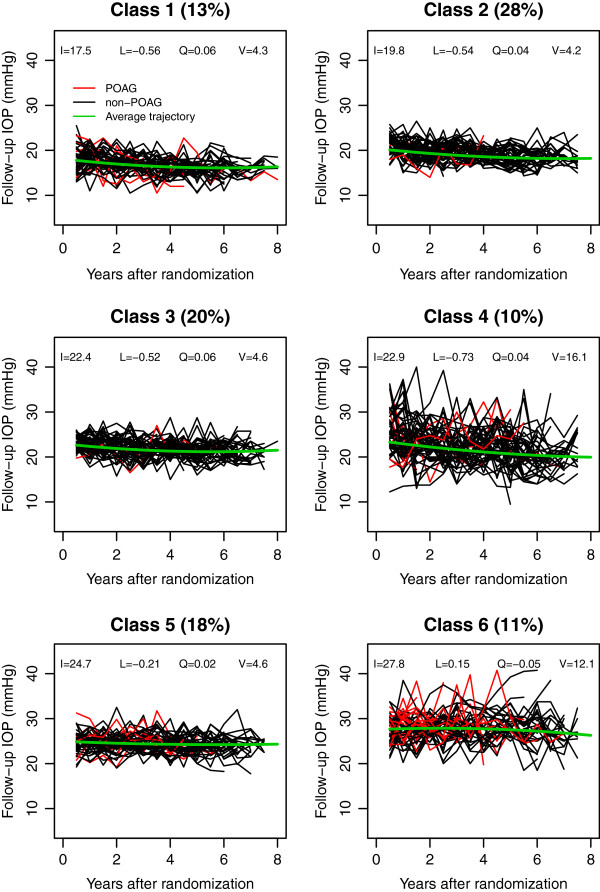
**Post-randomization IOP values for 50 subjects randomly selected from each of the 6 latent classes indentified in OHTS, where red lines represented subjects who developed POAG and the black lines were for those without POAG.** The class membership was based on the posterior probabilities from the optimal unconditional LCA, and the 4 parameters (I, L, Q, and V) in the plots represented the estimated initial level, the systematic linear and quadratic trend over time, and the variance of post-randomization IOP respectively.

Table 2 showed the fitting statistics of 7 competing LCAs for the OHTS and EGPS data. Based on the model-selection criteria, the IOP change in OHTS was best described by 6 distinct patterns (latent classes), which included 13%, 28%, 20%, 10%, 18% and 11% of the OHTS subjects respectively. Figure 2 showed the follow-up IOPs of 50 randomly selected subjects for each class. Most classes were distinguished primarily by the mean IOP levels. The only exceptions were classes 3 and 4. Classes 3 and 4 had similar average trajectories, but subjects in Class 4 showed a much larger variability. Figure 2 also indicated that the classes with higher mean level and/or larger variability had a higher risk of developing POAG. Table 3 reported the observed frequency of POAG in each class based on the most likely class membership. The hazard ratio (HR) and its 95% confidence interval (CI) of developing POAG in each class were also calculated using 1000 bootstrapping samples to account for the uncertainty in class membership. The results showed that the last 3 classes had significantly higher risk of developing POAG than the first 3 classes. For reasons that were not clear, however, subjects in Class 2 had the smallest risk though the subjects in Class 1 had the lowest mean follow-up IOP.

**Table 2:** Fitting statistics of 7 competing models that are only different in the number of latent classes

**# latent classes (G)**	**OHTS**			**EGPS**		
	**BIC**	LMR-LRT*	**Minimal class size**	**BIC**	**LMR-LRT**	**Minimal class size**
2	97097	<0.001	47%	39235	<0.001	44%
3	94219	0.002	24%	38395	0.001	14%
4	92922	0.609	14%	38109	0.005	11%
5	92107	0.003	13%	**37870**	**0.009**	**12%**
6	**91644**	**0.042**	**10%**	37760	0.452	9%
7	91289	0.147	7%	37682	0.060	5%
8	91045	0.406	6%	37608	0.011	4%

* Lo-Mendell-Rubin likelihood ratio test, with a smaller p-value favoring the G-class model over the model with G-1 classes (null hypothesis).

**Table 3:** Observed proportions of POAG, as well as estimated hazard ratios (HR) and 95% confidence intervals (CI) for POAG development in the unconditional LCAs for the OHTS and EGPS data, where the HR and 95% CI were based on 1000 bootstrapping samples to account for the uncertainty in the latent class membership

**Latent class**	**OHTS**			**EGPS**		
	**POAG%**	**HR**	**95% CI**	**POAG%**	**HR**	**95% CI**
1	5.9%	1.00	-	8.3%	1.00	-
2	3.9%	0.59	0.37 - 0.88	10.2%	1.28	0.76 - 2.06
3	4.3%	0.83	0.57 - 1.14	8.7%	1.13	0.73 - 1.65
4	10.1%	1.87	1.32 - 2.57	10.5%	1.40	0.85 - 2.18
5	11.4%	1.93	1.50 - 2.46	19.4%	2.66	1.92 - 3.69
6	31.2%	5.61	4.46 - 7.08			

**Figure 3:**
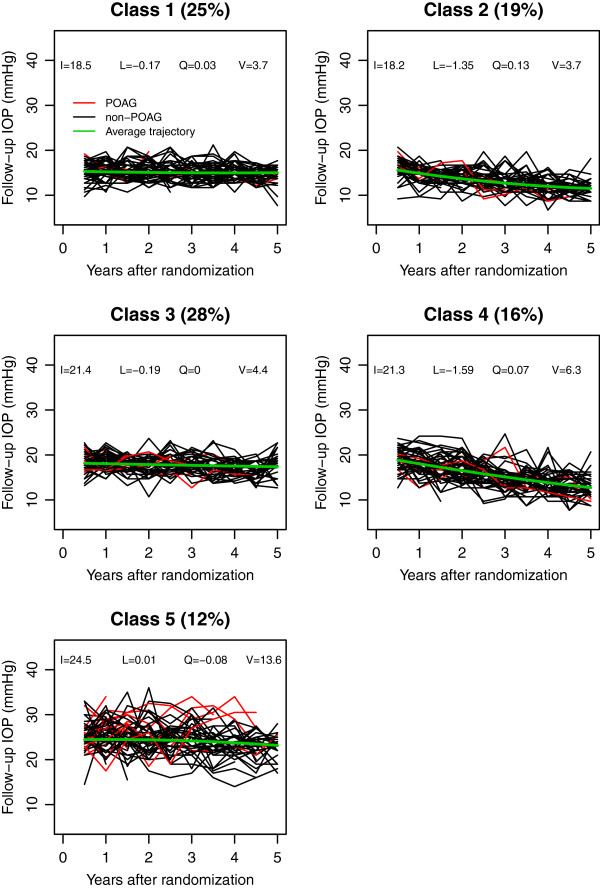
**Post-randomization IOP values for 50 subjects randomly selected from each of the 5 latent classes indentified in EGPS, where red lines represented subjects who developed POAG and the black lines were for those without POAG.** The class membership was based on the posterior probabilities from the optimal unconditional LCA, and the 4 parameters (I, L, Q, and V) in the plots represented the initial level, the systematic linear and quadratic trend over time, and the variance of post-randomization IOP respectively.

In EGPS, the IOP change was best fit by a 5-class LCA (Table 2). Figure 3 showed the post-randomization IOPs of 50 randomly selected subjects from each of the 5 classes, which included 25%, 19%, 28%, 16% and 12% of EGPS subjects respectively. Subjects in classes 1 and 2 started with similar initial follow-up IOP levels, but those in Class 2 showed a relatively rapid decrease over time. Similarly, subjects in classes 3 and 4 had similar initial levels, but subjects in Class 4 showed a relatively rapid decrease and subjects in Class 3 did not. All subjects in the first 4 classes presented similar magnitude of IOP variability. Subjects in Class 5 had the highest mean level and the largest variability, and they showed a significantly higher risk than the other 4 classes (Table 3).

Table 4 presented the distribution of treatment groups across latent classes in the OHTS and EGPS data respectively. In OHTS a great majority of subjects from treatment group fell into the first 3 classes, while in EGPS the distributions of treatment groups were rather similar across all latent classes.

**Table 4:** **Distribution of the randomization groups across latent classes, where the latent classes were based on the most likely *posterior class probability *from the optimal unconditional LCAs for the OHTS and EGPS data**

**Latent class**	**OHTS**			**EGPS**	
	**Observation**	**Treatment**	**Placebo**	**Treatment**	
1	15 (1.9%)	191 (24.0%)	113 (23.3%)	143 (29.4%)
2	67 (8.3%)	385 (48.4%)	69 (14.3%)	112 (23.0%)
3	226 (28.1%)	106 (13.3%)	162 (33.5%)	136 (27.9%)
4	55 (6.8%)	84 (10.6%)	64 (13.2%)	63 (12.9%)
5	279 (34.7%)	19 (2.4%)	76 (15.7%)	33 (6.8%)
6	163 (20.2%)	10 (1.3%)		
Total	805 (100%)	795 (100%)	484 (100%)	487 (100%)

### Conditional LCA

**Figure 4:**
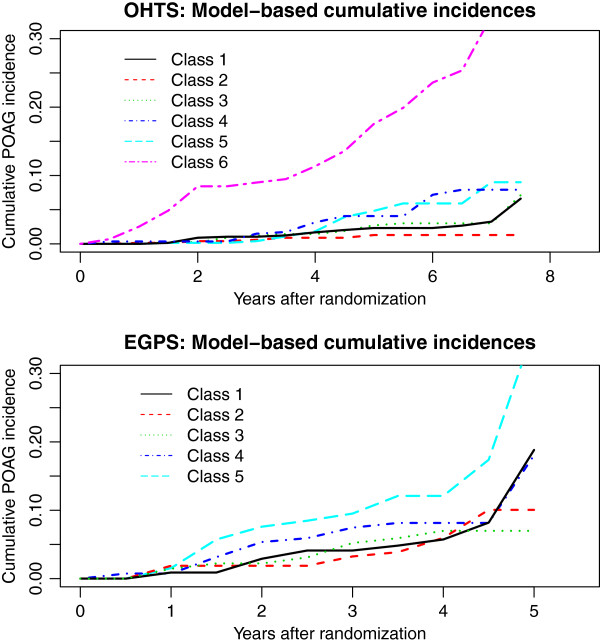
Predicted baseline cumulative incidence of POAG for each class, based on the conditional latent class analysis for the OHTS and EGPS data respectively.

A conditional model was constructed for OHTS and EGPS separately by adding the baseline factors as predictors and the time to POAG as the outcome to the optimal unconditional LCAs (Figure 1B). Since we had an adequate sample size in both studies, no variable-selection procedure was performed and all the baseline covariates (with the exception of dropping the variable race Black from EGPS because of lack of racial diversity) were included as predictors for both IOP change and the risk of developing POAG. Figure 4 presented the model-based predicted cumulative incidence for an “average” person with all baseline covariates being zero. After controlling for baseline covariates, different patterns of IOP change continued to be prognostic of POAG development. In both studies, the class with the highest mean level was most likely to develop POAG after adjusting for baseline IOP. In OHTS, subjects in Class 4 (with a moderate mean IOP and the largest variability) had similar risk as those in Class 5 (with a higher mean IOP and much less variability), but showed a higher risk than those in Class 3 (with the mean IOP comparable to Class 4 but with much less variability). In EGPS, the first 4 classes showed similar risk of developing POAG.

Table 5 presented the estimated parameters for within-class IOP trajectories, as well as the effects of baseline covariates on IOP classification and the risk of POAG development.

**Table 5:** Estimated parameters of the conditional LCAs in the OHTS and EGPS data

**OHTS**							
**Variables**	**Parameters for IOP change and the effects of covariates on class membership**						**Effects on POAG**
	**Class 1**	**Class2 (Ref.)**	**Class 3**	**Class 4**	**Class 5**	**Class 6**	
**Class Size IOP Change**	14.2%	27.1%	21.1%	9.1%	17.6%	10.9%	
I	17.58(0.20)^#^	19.83(0.21)^#^	22.30(0.21)^#^	22.82(0.79)^#^	24.74(0.22)#	27.70(0.28)^#^	-
L	−0.57(0.08)^#^	−0.53(0.06)^#^	−0.47(0.09)^#^	−0.95(0.30)^#^	−0.20(0.09)*	0.16(0.17)	-
Q	0.06(0.01)^#^	0.05(0.01)^#^	0.05(0.01)^#^	0.07(0.04)	0.02(0.02)	−0.05(0.03)	-
V	4.36(0.27)^#^	4.30(0.32)^#^	4.80(0.25)^#^	16.07(1.46)^#^	4.66(0.31)#	12.15(1.15)^#^	-
**Covariates**							
Intercept	−2.64(0.63)^#^		2.08(0.47)^#^	−0.06(0.47)	2.35(0.54)^#^	1.04(0.61)	-
TRT	1.60(0.66)^*^	-	−3.25(0.35)^#^	−2.19(0.63)^#^	−5.98(0.56)^#^	−6.46(0.70)^#^	0.16(0.29)
MALE	0.25(0.23)	-	−0.99(0.24)^#^	0.24(0.27)	−0.68(0.28)^*^	−0.22(0.30)	0.23(0.19)
RACEB	−0.10(0.27)	-	−0.37(0.30)	0.75(0.31)^*^	−0.91(0.34)^*^	0.05(0.37)	−0.05(0.24)
AGE	0.06(0.12)	-	−0.01(0.12)	0.08(0.16)	−0.05(0.14)	0.13(0.15)	0.18(0.09) ^*^
IOP0	−0.79(0.18)^#^	-	0.21(0.22)	1.03(0.36)^*^	0.89(0.18)^#^	1.73(0.22)^#^	−0.10(0.11)
CCT	−0.35(0.12)^*^	-	0.18(0.11)	−0.08(0.17)	0.14(0.13)	0.09(0.16)	−0.64(0.13)^#^
PSD	0.13(0.11)	-	0.04(0.11)	0.08(0.13)	0.17(0.13)	0.12(0.15)	0.23(0.10)^*^
VCD	0.06(0.11)	-	−0.08(0.12)	−0.15(0.17)	−0.10(0.13)	−0.09(0.15)	0.60(0.10)^#^
BB	−0.60(0.48)	-	−0.37(0.61)	- ^**^	−0.30(0.62)	−1.21(0.77)	0.19(0.57)
CHB	−0.19(0.37)	-	−0.32(0.42)	0.47(0.45)	−0.42(0.44)	−0.50(0.49)	0.09(0.31)
DM	−0.23(0.35)	-	0.22(0.32)	−0.71(0.45)	0.23(0.36)	0.64(0.40)	−1.67(0.53)^*^
HEART	0.70(0.44)	-	0.10(0.49)	0.43(0.56)	−0.09(0.56)	−0.48(0.73)	0.71(0.29)^*^
HBP	0.47(0.24)^*^	-	0.40(0.27)	0.06(0.34)	0.41(0.30)	0.66(0.33)^*^	0.08(0.22)
**EGPS**							
**Variables**	**Parameters for IOP change and the effects of covariates on class membership**					**Effects on POAG**	
	**Class1 (Ref.)**	**Class 2**	**Class 3**	**Class 4**	**Class 5**		
**Class Size IOP Change**	26.3%	20.1%	29.3%	12.9%	11.4%		
I	18.66(0.25) ^#^	18.24(0.18)^#^	21.24(0.20)^#^	21.85(0.47)^#^	24.33(0.34)^#^	-	
L	−0.34(0.14)^*^	−1.29(0.18)^#^	−0.25(0.11)^*^	−1.76(0.28)^#^	0.06(0.26)	-	
Q	0.05(0.03)	0.11(0.04)*	0.02(0.03)	0.02(0.07)	−0.08(0.07)	-	
V	3.79(0.23)^#^	3.75(0.25)^#^	4.59(0.24)^#^	7.12(0.85)^#^	12.17(1.32)^#^	-	
**Covariates**							
Intercept	-	−0.84(0.33)^*^	0.30(0.28)	−0.85(0.58)	−0.71(0.34)^*^	-	
TRT	-	0.36(0.27)	−0.65(0.23)^*^	−0.58(0.37)	−1.79(0.37)^#^	−0.01(0.21)	
MALE	-	−0.35(0.29)	0.14(0.23)	0.18(0.40)	0.38(0.33)	−0.24(0.22)	
Black	-	-	-	-	-	-	
AGE	-	−0.09(0.16)	0.01(0.13)	0.40(0.23)	0.45(0.20)^*^	0.16(0.10)	
IOP0	-	−0.61(0.24)^*^	0.82(0.17)^#^	1.24(0.24)^#^	1.79(0.23)^#^	0.11(0.13)	
CCT	-	−0.33(0.13)^*^	−0.14(0.12)	−0.43(0.15)^*^	0.09(0.15)	−0.36(0.12)^*^	
PSD	-	0.27(0.16)	−0.23(0.14)	−0.18(0.24)	−0.41(0.23)	0.18(0.09)^*^	
VCD	-	−0.13(0.17)	−0.03(0.13)	0.72(0.26)^*^	0.17(0.16)	0.46(0.12)^#^	
BB	-	−0.17(0.50)	−0.82(0.52)	- ^**^	−0.58(0.69)	−0.07(0.41)	
CHB	-	−0.10(0.56)	−0.97(0.52)	0.89(1.03)	−1.22(0.79)	−0.28(0.47)	
DM	-	−0.46(0.52)	0.12(0.45)	−1.32(1.27)	0.82(0.81)	−0.18(0.54)	
HEART	-	0.78(0.41)	0.11(0.44)	−0.83(0.77)	−0.79(0.61)	0.74(0.32)^*^	
HBP	-	0.02(0.36)	0.53(0.30)	−1.27(0.90)	0.11(0.54)	0.24(0.26)	

*: p < 0.05; #: p < 0.001; **: Not estimable due to zero count of beta blocker use in the given class.

### Effects of the baseline covariates on IOP classification

• To identify baseline predictors for IOP classification, we only focused on factors that were significantly associated with the high risk groups (Classes 4, 5, 6 in OHTS, and Class 5 in EGPS), while treating the lowest risk group (Class 2 in OHTS and Class 1 in EGPS) as the reference. In OHTS, treatment assignment and baseline IOP were two most important predictors for IOP classification. Subjects randomized to treatment group had a much lower chance of inclusion in the high risk groups (with OR = 0.11, 0.003, and 0.002 for Classes 4, 5, and 6, respectively), while these with a higher baseline IOP were more likely to be in the Classes 4, 5, or 6 (with OR = 2.80, 2.44, and 5.64 respectively). The results also showed that male subjects were less likely to be in Class 5 (OR = 0.51), the black subjects were more likely to be in Class 4 (OR = 2.12) but with a lower chance in Class 5 (OR = 0.40), and subjects with a history of hypertension were more likely in Class 6 (OR = 1.93). In EGPS, the results confirmed that treatment assignment (OR = 0.17) and baseline IOP level (OR = 5.99) were important predictors for Class 5. The result also showed that older age (OR = 1.57) was significantly associated with Class 5.

#### Effects of the baseline covariates on the risk of POAG development

• As expected, the effects of baseline covariates on the risk of developing POAG from the conditional LCA reached consistent conclusions as previous analyses using Cox models [17,25]. In OHTS, subjects with older age (HR = 1.20), higher PSD (HR = 1.26), large VCD (HR = 1.82), and history of heart diseases (HR = 2.03) had a higher risk of developing POAG, while thicker CCT (HR = 0.53) and history of diabetes (HR = 0.19) reduced the risk of developing POAG. Interestingly, despite marked differences between OHTS and EGPS in the patterns of IOP change, the EGPS confirmed 4 of the 6 predictors (except age and history of diabetes) identified in OHTS. In both studies, baseline IOP and treatment assignment were not significantly associated with POAG directly, but appeared to affect the risk indirectly through their strong influence on the classification of IOP change.

To explore the effect of follow-up IOP on the overall predictive accuracy of POAG, the 5-year cumulative POAG incidence was calculated for each individual using the formula (3). The overall predictive accuracy was summarized as C-index and calibration statistics (Model 1 in Table 6) [26]. For comparison, Table 6 also presented the C-index and calibration statistics from Cox models that only incorporated baseline predictors (Model 0). The results showed that adding post-randomization IOP considerably improved the predictive accuracy on POAG. In OHTS, for example, C-index increased from 0.778 to 0.821 by adding follow-up IOP. Given the fact that C-index from the baseline model was already high and there was little room for improvement, such an increase was substantial. An improvement in the C-index was also observed in EGPS though in a much smaller magnitude (from 0.706 to 0.719). The calibration statistics indicated that the model-based and observed survival probabilities were well agreed in both OHTS (*X*^2^ = 11.3) and EGPS (*X*^2^ = 7.0).

**Table 6:** Sensitivity analysis comparing the overall predictive accuracy (measured as C-index and Calibration Chi-square statistics) for LCAs with different model specifications

**Model**	**Model Features**	**C index**		**Calibration Chi-square**	
		**OHTS**	**EGPS**	**OHTS**	**EGPS**
0	Cox mode with baseline factors only	0.778	0.706	5.0	2.1
1	LCA with a quadratic within-class functional form	0.821	0.719	11.3	7.0
2	LCA with a linear within-class functional form	0.825	0.720	10.2	4.9
3	LCA with a constant within-class functional form	0.823	0.727	10.5	13.5

### Sensitivity analyses

As in all the statistical models, LCAs were inevitably based on certain assumptions. One assumption of our LCA was that the trajectories of IOP followed a quadratic functional form. It is known that the parameter estimates, class sizes, and interpretation of latent classes could be heavily influenced by the within-class distribution of longitudinal data [16]. In this section, first we assessed the sensitivity of risk prediction to different LCA specifications. Table 6 presented the C-index and calibration statistics for LCAs after removing the quadratic term (Model 2) or removing both quadratic and linear terms (Model 3). The results showed that LCAs had a robust performance in terms of predictive accuracy for POAG development.

**Figure 5:**
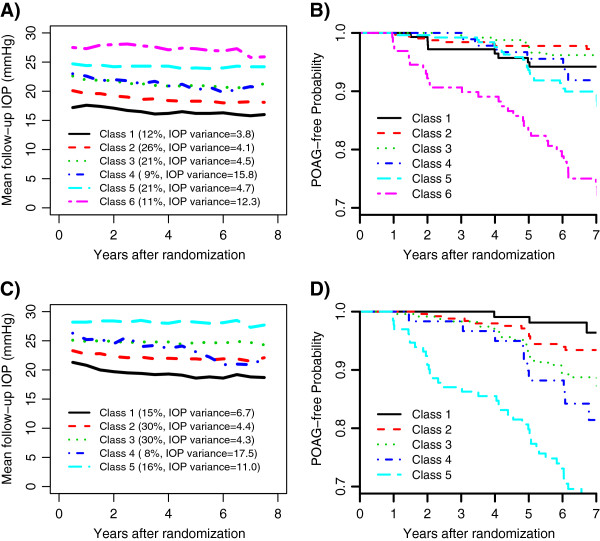
**Sensitivity analyses of latent class models in the OHTS data.** Plots **A** and **B**: the observed mean IOP during follow-up visits of the latent classes and the corresponding Kaplan-Meier POAG-free curves in the non-Black participants; Plots C and D: the observed mean IOP during follow-up visits of the latent classes and the corresponding Kaplan-Meier POAG-free curves in the untreated participants.

Next, two additional sensitivity analyses were performed in the OHTS data, one excluding participants with Black race and the other only using participants randomized to the observation group. The IOP change in the non-Black was best described by 6 distinct classes, while the LCA in the untreated participants identified 5 classes. Figures 5A and 5C showed the observed mean IOP of latent classes in the non-Black and untreated participants, respectively. Although most classes were distinguished primarily by the mean IOP, each LCA identified a subgroup of participants (Class 4) who had a moderate IOP mean but with the highest IOP variability. More interestingly, the participants from Class 4 in both LCAs showed relatively higher risk of POAG development than those with a comparable mean IOP (Figures 5B and 5D).

Finally, our LCA also made an implicit assumption that the baseline covariates influenced the IOP change exclusively through their effects on the class membership (i.e., no direct effects on the within-class growth parameters). The validity of this assumption can be checked by comparing the conditional LCAs with the unconditional models. The assumption violation is often signified by a dramatic shifting in the meaning and size of latent classes when the baseline predictors are added to the unconditional LCA [16]. Based on the estimated class-specific parameters (Table 5, Figures 2 and 3), this assumption was well satisfied in both studies.

## Discussion

In recent years, one of the hot topics in glaucoma research has been the effect of IOP fluctuation on POAG. Although more and more studies have confirmed that a decrease in the mean IOP level can reduce the risk of developing POAG, the findings from major prospective clinical trials about the impact of IOP fluctuation on POAG remain controversial [25,27-30]. In this paper, we analyzed the post-randomization IOPs from OHTS and EGPS taking a latent class analysis (LCA) approach. The LCA allows us to identify distinct patterns of IOP change over time and then associates the changes in IOP with the risk of POAG. The results from both studies showed that different patterns of IOP change could markedly affect the risk of POAG (irrespective of their baseline, pre-randomization IOP levels). In OHTS, the change in IOP was best described by 6 distinct patterns. The model identified a subset of participants in whom IOP variability also played an important role in predicting POAG. This subgroup showed the highest IOP variability and had a higher risk than those with a comparable IOP mean. Comparing to the reference class, these participants were less likely from treatment group (OR = 0.11), more likely self-classified as being black (OR = 2.12), and had relatively higher baseline IOP (OR = 2.80). However, the subgroup only accounted for about 10% of the OHTS sample, and this may partially explain our finding that IOP variability was an independent risk factor in the OHTS but had little impact on the overall predictive accuracy for POAG (manuscript in progression). In a sensitivity analysis using the non-Black participants, the LCA identified similar patterns of IOP change as in the whole OHTS dataset. This result was consistent with a tree-based model in the OHTS-EGPS meta-analysis which showed that race was no longer an important predictor for POAG development after considering other risk factors [17]. In EGPS, LCA identified 5 distinct latent classes and confirmed that those subjects with the highest mean IOP were most likely to develop POAG. However, it failed to disentangle the effect of fluctuation from mean because these participants with the highest mean level also had the largest IOP variability. Interestingly, despite the marked differences between EGPS and OHTS in the treatment intervention and magnitude of IOP lowering achieved, both studies showed that adding IOP change into the baseline model improved the overall predictive accuracy for POAG development.

Conventionally the change of longitudinal data is described using linear mixed models with random coefficients [31]. Though the mixed model recognizes the heterogeneous nature of the data by allowing each individual to have his/her own intercept and slope, it assumes that all individuals come from a single population and uses an average trajectory for the entire population. A LCA analyzes data from a rather different perspective. The model approximates the unknown heterogeneity in the distribution of longitudinal outcome using a finite number of polynomial functions each describing a unique subpopulation [14,32]. It classifies individuals into distinct groups based on the patterns of longitudinal outcome, so that individual within a group are more similar than those between different groups. This LCA possesses some unique advantages as comparing to conventional methods. First, the model lends itself directly to a set of well characterized subpopulations and also provides a formal statistical procedure to determine the appropriate number of subpopulations. It thus enables the discovery of unexpected yet potentially meaningful subpopulations that may be otherwise missed with conventional methods. Second, the method permits one to relate the developmental patterns of longitudinal data to its antecedents (predictors or covariates) and consequences (clinical outcomes), and thus allows estimation of both direct and indirect (via longitudinal data) effects of a covariate on the distant outcome [16,23]. Finally, the recent advances of the dual trajectory modeling also allow investigators to assess the joint evolution of multiple longitudinal processes, which may evolve contemporaneously or over different time periods [32].

LCA also provides an attractive alternative for making prediction with time-dependent covariates [21,22]. A LCA takes a joint modeling approach to assess the association between longitudinal and survival data and thus uses information more efficiently, resulting less biased estimates. Unlike the conventional joint models that assess the association via shared random effects [19,33,34], a LCA relates the longitudinal process to survival process by latent classes and assumes the two stochastic processes independent given the class membership [22]. Therefore, neither time-dependent covariates nor random effects of the longitudinal data are needed in the survival sub-model. Such a model specification will avoid the intensive computation to obtain the random effects for new subjects and hence facilitates a real-time individualized prediction [21]. The key to build an accurate prediction in a LCA setting is to have a reliable classification given the observed data. Generally speaking, the more the available serial biomarker readings, the more reliable a classification is. To this consideration, the impact of follow-up IOP on POAG may be over-estimated in OHTS because an average length of 6.5-year IOP readings was used to calculate the 5-year POAG-free rate. To solve this dilemma, which is rather common in all predictions involving time-dependent covariates, one of the most frequently used approaches in medical literature is a landmark analysis that consists of fitting a serial of survival models only to the subjects still at risk, that is, computation of the predictive distribution at a certain time given the history of event and covariates until that moment [35]. In a LCA setting, such a dynamic prediction can be conveniently implemented because the conditional survival probability at any time can be calculated analytically from a single LCA once the parameters are estimated [21].

Despite its advantages, the LCA has several limitations. First, the computational load of LCA can be high, especially for models with complexity structures. In OHTS data (N = 1600), for example, it ran less than 10 minutes for an unconditional 6-class LCA, but it took more than 30 minutes to develop the full conditional model. Because of the exploratory nature of data analysis with LCA, the cumulative time can be substantial. For this consideration, in practice the best LCA model is often constructed taking a two-step approach as in this paper. Another issue in LCA is that the log-likelihood function may end up at local rather than global maxima. Fortunately this issue has been taken into consideration by the statistical package Mplus which automatically uses 10 sets of randomly generated starting values for estimation. The program also allows investigators to rerun and compare the estimates from user specified starting values if necessary [23].

## Conclusion

LCA provides a useful alternative to understand the interrelationship among the baseline covariates, the change in follow-up IOP, and the risk of developing POAG. The inclusion of post-randomization IOP can improve the predictive ability of the original prediction model that only included baseline risk factors.

## Abbreviations

IOP: Intraocular pressure; POAG: Primary open-angle glaucoma; LCA: Latent class analysis; OHTS: The ocular hypertension treatment study; EGPS: The european glaucoma prevention study; HR: Hazards ratio; OR: Odds ratio; CCT: Central corneal thickness; PSD: Pattern standard deviation; VCD: Vertical cup/disc ratio.

## Competing interests

The authors declare that they have no competing interests.

## Authors' contributions

FG, JPM, JAB and MOG conceived the study. FG and JAB carried out the data analysis. FG and MOG drafted the first version of the manuscript. All authors contributed to the critical review and approved the final version.

## Pre-publication history

The pre-publication history for this paper can be accessed here:

http://www.biomedcentral.com/1471-2288/12/151/prepub
